# Modelling malaria elimination on the internet

**DOI:** 10.1186/1475-2875-10-191

**Published:** 2011-07-14

**Authors:** Richard J Maude, Sompob Saralamba, Adrian Lewis, Dean Sherwood, Nicholas J White, Nicholas PJ Day, Arjen M Dondorp, Lisa J White

**Affiliations:** 1 Mahidol-Oxford Tropical Medicine Research Unit (MORU), Faculty of Tropical Medicine, Mahidol University, Bangkok, Thailand; 2 Centre for Tropical Medicine, Nuffield Department of Clinical Medicine, Churchill Hospital, University of Oxford, Oxford, UK; 3 iSynch-D, 11/3, Soi Sirisuk, Kwang Sasennok, Khet Huaykwang, Bangkok, Thailand

## Abstract

**Background:**

Unprecedented efforts are underway to eliminate malaria. Mathematical modelling can help to determine the optimal strategies for malaria elimination in different epidemiological settings. This is necessary as there is limited scope for expensive and time-consuming field studies and failure of planned elimination strategies is likely to discourage ongoing investment by funders. However, there has been very little modelling of malaria elimination and little direct involvement of policymakers in its development. There is thus an urgent need for user-friendly and accessible models purpose-designed in collaboration with policymakers to answer pertinent questions arising from the field.

**Results:**

An internet site is presented with a simple mathematical modelling platform for population level models of malaria elimination. It is freely accessible to all and designed to be flexible so both the platform and models can be developed through interaction with users. The site is an accessible introduction to modelling for a non-mathematical audience, and lessons learned from the project will help inform future development of mathematical models and improve communication of modelling results. Currently it hosts a simple model of strategies for malaria elimination and this will be developed, and more models added, over time. The iterative process of feedback and development will result in an educational and planning tool for non-modellers to assist with malaria elimination efforts worldwide.

**Conclusions:**

By collaboration with end users, iterative development of mathematical models of malaria elimination through this internet platform will maximize its potential as an educational and public health policy planning tool. It will also assist with preliminary optimisation of local malaria elimination strategies before commitment of valuable resources.

## Background

Mathematical modelling has great potential as a tool to help guide efforts towards malaria elimination [1]. Different combinations of interventions are required for different epidemiological settings. However, there are limited data available to policymakers to inform their decisions on which strategies to employ. Mathematical modelling combines mechanistic understanding with available data from multiple sources to make predictions. It could potentially be used for preliminary evaluation of different strategies for malaria elimination in different epidemiological contexts much more rapidly and at lower cost than is possible through trial and error in the field [1]. Modelling is particularly useful where a field study cannot be done as is the case with large-scale elimination programmes for which it is desirable to get the strategy right first time. There is a risk that the current unprecedented commitment of financial resources by international agencies will not be sustained if attempts at elimination are perceived to be failing.

Although modelling is beginning to be used for this purpose [2], it is often inaccessible to the majority of those planning malaria elimination efforts. Much of the modelling currently undertaken remains in the hands of the specialist research community and is presented as selected results in the scientific literature. Although recent modelling studies have provided useful generally applicable insights, interaction with policymakers at the level of individual nations is rare. This remains a major barrier to realising the potential of mathematical modelling in this area. In addition, awareness must be raised and training provided to policymakers in order for them to access mathematical modelling effectively for their individual circumstances.

One solution to this problem is to make models accessible to all via the internet. Several such models are available for malaria [3-5] and more are in development. One inherent problem with this method is that to remain user-friendly, websites need to run quickly. It is technically difficult to incorporate a complex model into a website and maintain usability as such models tend to be processor-intensive and thus run slowly. One solution to this is to present a range of pre-prepared modelling results from model runs carried out offline. This has the drawbacks that the range of possible scenarios is limited and the user has less scope for exploring the behaviour of the model, for example with interacting interventions. Another approach has been to offer the model as a downloadable and installable application e.g. the Malaria Tools project (currently limited to a population of up to 1000 people and certain pre-specified scenarios) [3]. This has the advantages that it uses the native processing power of the user's computer and can be run offline. However, not all users want or are able (many workplace security systems do not allow it) to install third party code and to update such a model requires a new version of the software to be installed each time.

An alternative approach is to incorporate the model code into a website where it can be run in situ in the web browser and easily adjusted and expanded by the designer. Several approaches to this are being attempted. By using cloud computing, the model can be quite complex. This is being trialled at malariacontrol.net where the computing power of large numbers of volunteers is accessed over the internet [4]. However, in its current form, the input parameters and model runs are set by the malariacontrol.net team and outside users cannot run the models themselves. Another approach has been to restrict the model to detailed exploration of the potential impact of a single intervention, e.g. intermittent preventive treatment in infants [5].

This paper describes a flexible and user-friendly website with an online mathematical model of malaria elimination that is being developed interactively with end-users. There is a trade-off between user-friendliness and model complexity, and hence the underlying structure of the model is relatively simple [6]. The aim is for this to be an accessible introduction to mathematical modelling for those less familiar with the discipline and ultimately, by further development, a simple tool with which malaria control programmes can explore the potential impact of strategies being considered for deployment. The current model is based on a previously published framework and there is a detailed description elsewhere [6]. This methodology paper focusses particularly on the web platform.

## Results

### Software suite

**Figure 1:**
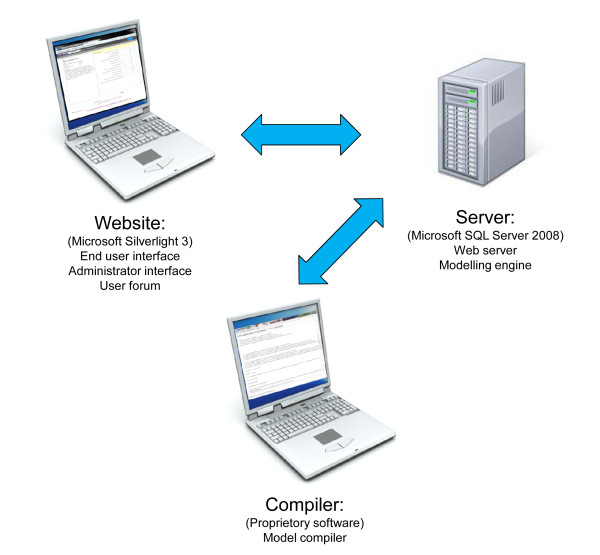
**Structure of software suite**.

A dedicated software suite was developed to present ordinary differential equation models in a user-friendly format on the internet. This uses the .NET framework (Microsoft™) and consists of a number of proprietary components developed specifically for this project (Figure 1): 1) database using SQL Server 2008 (Microsoft™), and a modelling engine server including an ordinary differential equation solver using RK4, 2) client application for preparation and entering the modelling code in a simple intuitive format with a compiler to convert the code to a C# native code assembly and 3) a website that end users and administrators can access via the Silverlight 3 (Microsoft™) application and including a user forum. The website is described in more detail below followed by information on the current model.

### Website

**Figure 2:**
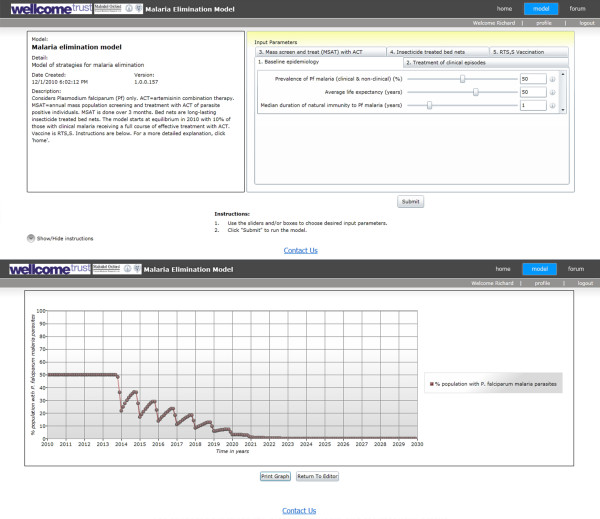
**Screenshots of the malaria elimination model internet site**. The upper portion of the figure is the parameter input screen with sliders to select values for different categories of parameters. The lower portion is an example of the model output.

The website can be accessed at http://www.tropmedres.ac/elimination[7]. Screenshots are shown in Figure 2. This is a purpose-designed portal for the presentation of population dynamic mathematical models in an accessible and user-friendly format. It has the advantages that users have direct access to the model interface over the internet allowing them to adjust input parameters, enter their own data if desired, and run the model themselves. As the programme runs very rapidly, it is possible to try a wide range of modelled scenarios and explore model behaviour. There are three levels of user access: anonymous, registered and administrative. Users are encouraged to register their details as this allows them access to a more sophisticated interface with a broader range of input parameters, plus the ability to enter comments and interact with the development team on the forum. With administrative access, changes can be made to the model code, including its parameters and structure, and the forum administered, all from any location with internet access.

### Mathematical model

The website currently hosts a simple population level mathematical model created to examine the elimination potential of all the current major malaria control interventions, alone and in combination, in a variety of settings. This model uses a previously published structure with a compartmental deterministic framework based on ordinary differential equations. Details and modelling results have been published elsewhere [6]. It should be noted that although it is able to reproduce the general results of more sophisticated models, in its current form it is not intended for use in real-world planning scenarios. The model was entered into the compiler and converted for use on the website. After a period of internal beta testing it was first made available online on 2^nd ^December 2010 [8]. The model structure and parameters are under ongoing development and refinement in collaboration with users of the website who can communicate with the site development team by email (via the 'Contact Us' link) and, when registered, with other users via a recently added online forum (link at the top right). The current iteration of the model can be accessed online [8] and a detailed User Guide is available to download in pdf format [9].

## Discussion

Mathematical modelling has an important contribution to make to planning malaria elimination [10] but has been under-utilized to date. This project provides an accessible and user-friendly platform for presenting mathematical models on the internet that is suitable for use by non-modellers. In the initial phase, a simple model of malaria elimination is presented that is not suitable for detailed planning of real-world strategies. As is the ideal for mathematical modelling, this will be developed as an ongoing iterative process of collaboration with the end users of the modelling results to produce modelling tools that are increasingly useful and relevant. Although it currently hosts a single model, multiple different models will be available on the site designed to answer specific and pressing questions from policymakers. The development team will work in collaboration with users to update and refine the platform to maximize its potential and if necessary to assist non-mathematicians with the interpretation of the model results. It is hoped that an online community can be built and by continuous refinement develop the platform into a usable tool to help plan local strategies for malaria elimination. Readers are encouraged to try the platform for themselves and invited to get involved in what is hoped will be a useful contribution to malaria elimination efforts worldwide.

## Conclusions

A free, internet-based, user-friendly and interactive platform for mathematical models of malaria elimination is presented. This platform will be developed and refined in collaboration with users to maximize its potential as an educational and public health policy planning tool. This will assist with preliminary optimisation of local malaria elimination strategies before commitment of valuable resources.

## Competing interests

The authors declare that they have no competing interests.

## Authors' contributions

RJM and LJW created the mathematical models, developed the website and wrote the paper. AL wrote the compiler software. AL, SS & DS assisted with website development. AL, SS, DS, NPJD and AMD reviewed the manuscript. LJW supervised the work. All authors have read and approved the final manuscript.
